# Vertical Integration and Care Experiences Among Medicare Advantage Beneficiaries

**DOI:** 10.1001/jamanetworkopen.2024.38994

**Published:** 2024-10-17

**Authors:** Geronimo Bejarano, Andrew Ryan, Amal Trivedi, David J. Meyers

**Affiliations:** 1 Department of Health Services, Policy, and Practice, Brown University School of Public Health, Providence, Rhode Island; 2 Providence Veterans Affairs Medical Center, Providence, Rhode Island

## Abstract

**Importance:**

Medicare Advantage (MA) beneficiaries are increasingly enrolling in integrated MA plans. Legacy-integrated plans share unique features that may differ from newer integrated MA plans. It is unclear whether integrated and legacy-integrated MA plans are associated with a better beneficiary care experience compared with non–legacy-integrated and nonintegrated MA plans.

**Objective:**

To assess whether enrollment in integrated, legacy-integrated, non–legacy-integrated, and nonintegrated MA plans is associated with better beneficiary care experiences.

**Design, Setting, and Participants:**

This cross-sectional study included MA beneficiaries who responded to Consumer Assessment of Healthcare Providers and Systems (CAHPS) surveys conducted annually between 2015 and 2019. Data analysis was performed between October 1, 2023, and July 31, 2024.

**Exposure:**

Medicare Advantage plan integration status, categorized as integrated, legacy-integrated, non–legacy-integrated, and nonintegrated.

**Main Outcomes and Measures:**

The CAHPS surveys assessed 9 care experience measures. To assess differences in care experience measures, mixed linear regression analyses were performed, adjusting for demographic characteristics, plan random effects, and state fixed effects.

**Results:**

The sample consisted of 857 695 respondents. Their mean (SD) age was 72.6 (10.3) years, and 58.1% were women. A total of 12.7% of respondents were Black, 10.7% were Hispanic, and 71.0% were White. Compared with respondents in nonintegrated MA plans, respondents in integrated MA plans were younger (mean [SD] age, 72.7 [10.3] vs 72.2 [10.3] years, respectively) and were less likely to be Black (13.1% vs 11.2%, respectively), female (58.4% vs 57.2%, respectively), and partially dual eligible (8.7% vs 6.2%, respectively). Integrated MA plans were associated with meaningfully better mean ratings only of customer service (1.6 points [95% CI, 1.1-2.1]) and health plan (1.0 points [95% CI, 0.6-1.5]) compared with nonintegrated MA plans. Legacy-integrated MA plans were associated with meaningfully better mean ratings of drug plan (3.7 points [95% CI, 2.4-5.0]), health plan (3.1 points [95% CI, 1.7-4.5]), customer service (2.8 points [95% CI, 1.3-4.4]), getting appointments and care quickly (2.6 points [95% CI, 1.2-4.0]), health care quality (1.9 points [95% CI, 1.0-2.7]), physicians who communicate well (1.1 points [95% CI, 0.4-1.8]), and care coordination (1.1 points [95% CI, 0.4-1.9]) compared with nonintegrated MA plans.

**Conclusions and Relevance:**

In this study, integrated MA plans were not associated with better beneficiary care experiences compared with nonintegrated MA plans. Legacy-integrated MA plans were associated with higher ratings on all care experience measures compared with both non–legacy-integrated and nonintegrated MA plans. Monitoring of integrated MA plans is needed to assess whether they are adding meaningful value to MA beneficiaries and to determine their effects on the health care system.

## Key Points

**Question:**

Does beneficiary care experience differ among integrated, legacy-integrated, non–legacy-integrated, and nonintegrated Medicare Advantage (MA) plans?

**Findings:**

In this cross-sectional study of 857 695 survey respondents, enrollment in integrated MA plans was associated with meaningfully higher ratings of only 2 (customer service and health plan) of 9 care experience measures compared with nonintegrated MA plans. Enrollment in legacy-integrated MA plans was associated with higher ratings of all 9 care experience measures compared with enrollment in non–legacy-integrated and nonintegrated MA plans.

**Meaning:**

These findings suggest that only legacy-integrated MA plans are associated with meaningfully better beneficiary care experiences compared with nonintegrated MA plans.

## Teaser

This cross-sectional study uses beneficiary survey data to explore the association between care experience and enrollment in integrated, legacy-integrated, non–legacy-integrated, and nonintegrated Medicare Advantage plans.

## Introduction

Enrollment in Medicare Advantage (MA) plans has exceeded traditional Medicare and is expected to continue increasing.^1^ Medicare Advantage plans are paid through a capitated model incentivizing them to manage the care of their beneficiaries using care coordination, networks, and prior authorization.^2^ There is a growing trend in which MA plans and health systems are vertically integrating to align incentives so they can own the “whole dollar” from the capitated payments.^3,4,5^ These integrated MA plans are created either when a health system starts its own MA plan or when an MA plan acquires practitioners to deliver care for their beneficiaries.^6,7^ This approach differs from horizontal integration, which occurs when health systems purchase another health system or MA plans purchase another MA plan.^8^ For health systems, vertical integration creates the potential to increase revenues through setting health insurance premiums, managing MA care and capitated payments, and collecting bonuses from the MA quality bonus program.^9,10,11^ Yet vertical integration requires health systems to take on the many challenging functions of private insurers.

There is important heterogeneity within integrated MA plans that may affect the care experience.^12,13^ Although vertical integration can be most easily understood as a health system and an MA plan having a shared parent company, the form of integration can vary in several ways. Some integrated MA plans may have more clinical integration, which can allow for ease of care coordination and assurance of coverage for treatment. Meanwhile, other integrated MA plans may be vertically integrated financially but have limited integration in care or claims processing. Acknowledging the importance of heterogenous integration, integrated MA plans can be divided into roughly 2 groups: legacy- and non–legacy-integrated MA plans. Legacy plans, including Kaiser Permanente, Intermountain Health, and Geisinger, were formed decades ago, well before the recent rise in MA enrollment. These plans have been proponents of performance measurement and benchmarking, were early adopters of electronic health record technology, and have tended to employ physicians with salary-based compensation.^14,15,16^ On the other hand, non–legacy-integrated MA plans, including those in academic medical centers, have formed more recently around MA business.^17^ These nascent plans may have decentralized management structures across the insurance and clinical functions, reducing the likelihood of improved care coordination and patient experience. Additionally, these plans may not have enrolled a sufficient volume of beneficiaries, often deemed as the reason for legacy-integrated MA plan success. These new plans may be less experienced in attracting healthier beneficiaries who are more likely to report better experience with care.^12,18^

Although frequently cited as a justification for integration, the effect of vertical integration on the care experience is unknown. Proponents of vertical integration highlight the potential of increased efficiency through care coordination leading to better care experience.^6^ Meanwhile, critics of vertical integration argue that the profit motives and increased market power will lead to higher premiums and more inappropriate spending in MA without improvement in the care experience.^19,20^ Due to a lack of data on vertical integration status, current evidence on the effect of vertical integration on MA plans has been limited. Prior studies have found that integrated MA plans are associated with better star rating quality and more supplemental benefits, which may improve the beneficiary care experience; however, these plans also have higher premiums and cost sharing, which may decrease the beneficiary care experience.^3,7^ In this context, we analyzed MA Consumer Assessment of Healthcare Providers and Systems (CAHPS) survey data to compare care experiences among beneficiaries in integrated, legacy-integrated, non–legacy-integrated, and nonintegrated MA plans.

## Methods

In this cross-sectional study, we analyzed respondent-level MA CAHPS survey data from 2015 to 2019. The MA CAHPS survey aims to assess the beneficiary care experience and is conducted annually on a sample of approximately 600 MA beneficiaries per contract.^21^ The Centers for Medicare & Medicaid Services uses the CAHPS data to assess contract performance. The Brown University Institutional Review Board deemed this study exempt from review, with a waiver of informed consent due to the use of deidentified data. The study followed the Strengthening the Reporting of Observational Studies in Epidemiology (STROBE) reporting guideline.

### Data Sources

We created a longitudinal dataset of the integration status of MA plans using publicly available MA contract information by evaluating whether individual contracts were integrated with a health system. To construct our dataset of integrated MA contracts, we assessed every unique MA contract between 2015 and 2019 to classify each contract as either integrated or nonintegrated (eMethods in Supplement 1). Beneficiaries were considered to be in an integrated MA plan if they enrolled in an MA plan the year of or after integration between MA plan and health system. We linked the integrated MA data to the Medicare Beneficiary Summary File to classify MA beneficiaries as either enrolled in integrated MA plans or not and for beneficiary characteristics. We considered MA beneficiaries who were in Kaiser Permanente, Geisinger, or Intermountain Health plans to be in legacy-integrated MA plans. We chose these plans as legacy-integrated MA plans because they integrated before the rise of MA enrollment, have the largest portion of integrated MA plan beneficiaries, and have reported unique attributes (eg, a critical number of beneficiaries and salaried employed practitioners, and leveraging integration to hold practitioners accountable for savings through quality and technological improvement) to their success in integrated care.^12,13,15,22^ Beneficiaries in any other integrated MA plan were considered to be enrolled in non–legacy-integrated MA plans. We included beneficiaries if they were enrolled in the MA plan in January of that year. Finally, we merged in the outcome measures of beneficiary care experiences from the MA CAHPS and only included beneficiaries who had a survey with no missing data and whose survey response was complete for analysis and reporting according to the survey completion variable as suggested by MA CAHPS documentation.^21^

Race and ethnicity data were self-reported in the survey and were categorized as American Indian or Alaska Native, Asian, Black, Hispanic, White, or other race or ethnicity (which comprised any other race or ethnicity not included in the options listed). These data were collected because of potential differences in care experience by race and ethnicity that arise from social inequities.

### Study Outcomes

We calculated 9 outcome measures of beneficiary care experiences, including 6 composite measures (getting needed care, getting appointments and care quickly, physicians who communicate well, care coordination, customer service, and getting needed prescription drugs) and 3 single-item measures (rating of health plan, health care quality, and drug plan). Each outcome measure was scored from 0 to 100, with 100 representing the highest possible score. These outcome measures have been used in prior literature to compare care experiences between plans, with 1-, 3-, and 5-point between-group differences considered small, medium, and large, respectively.^23,24,25,26^ The eMethods in Supplement 1 further detail the outcome measures.

### Statistical Analysis

We compared the association of enrollment in an integrated MA plan (integrated MA plan vs nonintegrated MA plan, legacy-integrated MA plan vs nonintegrated MA plan, and legacy MA plan vs non–legacy-integrated MA plan) with each measure using respondent-level mixed linear regressions, adjusting for age, race and ethnicity, sex, dual eligibility with Medicaid, whether a proxy filled out the survey, health status (poor, fair, good, very good, or excellent), survey language, plan random effects, and state fixed effects. We report the adjusted mean (SE) of each outcome measure for beneficiaries in integrated and nonintegrated MA plans. We tested the assumptions of linear regression and found no troubling collinearity or assumption violations. *P* < .05 (2-sided) was considered statistically significant. As a sensitivity analysis, we excluded beneficiaries who were in Kaiser Permanente integrated MA plans, which comprise the largest group of integrated MA plans.

Statistical analyses were conducted using Stata, version 18 (StataCorp LLC). Data were analyzed between October 1, 2023, and July 31, 2024.

## Results

Our sample consisted of 857 695 MA CAHPS respondents, 23.7% of whom were enrolled in an integrated MA plan (Table). Their mean (SD) age was 72.6 (10.3) years; 58.1% were women and 41.9% were men. A total of 0.3% of respondents were American Indian or Alaska Native, 3.3% were Asian, 12.7% were Black, 10.7% were Hispanic, 71.0% were White, and 2.0% were of other race or ethnicity. Of the 857 695 respondents, 47.8% were in legacy-integrated plans, 41.9% were in non–legacy-integrated plans, and 38.6% were in nonintegrated MA plans (eTable 2 in Supplement 1). Overall, most respondents were nondual-eligible (70.2%), did not use a proxy to complete the survey (88.3%), reported good health (36.4%), and completed the survey in English (95.4%). A lower proportion of MA beneficiaries in integrated plans responded to the MA CAHPS survey (58.7%) compared with those in nonintegrated plans (62.4%). Compared with respondents in nonintegrated MA plans, a lower proportion of respondents in integrated MA plans were Black (11.2% vs 13.1%), female (57.2% vs 58.4%), and partially dual eligible (6.2% vs 8.7%).

**Table.** Demographic Characteristics of MA Beneficiaries Stratified by Integration Type^a^

Characteristic	All beneficiaries (N = 857 695 [100])	MA plan type^b^			
		All integrated (n = 203 054 [23.7])	Nonintegrated (n = 654 641 [76.3])	Legacy-integrated (n = 25 181 [12.4])	Non–legacy-integrated (n = 177 873 [87.6])
Age, mean (SD)	72.6 (10.3)	72.2 (10.3)	72.7 (10.3)	74.5 (8.1)	71.9 (10.5)
Race and ethnicity					
American Indian or Alaska Native	2665 (0.3)	849 (0.4)	1816 (0.3)	35 (0.1)	814 (0.5)
Asian	28 674 (3.3)	8241 (4.1)	20 433 (3.1)	1019 (4.1)	7222 (4.1)
Black	108 549 (12.7)	22 668 (11.2)	85 881 (13.1)	2154 (8.6)	20 514 (11.5)
Hispanic	91 884 (10.7)	22 035 (10.8)	69 849 (10.7)	1813 (7.2)	20 222 (11.4)
White	609 097 (71.0)	144 471 (71.1)	464 626 (71.0)	19 495 (77.4)	124 976 (70.3)
Other^c^	16 826 (2.0)	4790 (2.4)	12 036 (1.8)	665 (2.6)	4125 (2.3)
Sex					
Female	498 373 (58.1)	116 249 (57.2)	382 124 (58.4)	14 156 (56.2)	102 093 (57.4)
Male	359 322 (41.9)	86 805 (42.8)	272 517 (41.6)	11 025 (43.8)	75 780 (42.6)
Medicaid eligibility					
Full dual	186 400 (21.7)	47 431 (23.4)	138 969 (21.2)	1062 (4.2)	46 369 (26.1)
Partial dual	69 529 (8.1)	12 556 (6.2)	56 973 (8.7)	664 (2.6)	11 892 (6.7)
Nondual	601 763 (70.2)	143 064 (70.4)	458 699 (70.1)	23 455 (93.2)	119 609 (67.2)
Proxy to complete survey					
Yes	97 961 (11.7)	21 692 (10.9)	76 269 (12.0)	1814 (7.3)	19 878 (11.4)
No	738 654 (88.3)	176 818 (89.1)	561 836 (88.0)	23 009 (92.7)	153 809 (88.6)
Health status					
Poor	49 882 (5.9)	11 537 (5.8)	38 345 (6.0)	839 (3.4)	10 698 (6.1)
Fair	196 828 (23.4)	45 260 (22.7)	151 568 (23.6)	4130 (16.8)	41 130 (23.5)
Good	306 827 (36.4)	71 603 (35.9)	235 224 (36.6)	9302 (37.8)	62 301 (35.6)
Very good	219 464 (26.1)	54 180 (27.1)	165 284 (25.7)	7868 (31.9)	46 312 (26.5)
Excellent	68 763 (8.2)	17 039 (8.5)	51 724 (8.1)	2500 (10.2)	14 539 (8.3)
English survey language					
Yes	818 164 (95.4)	192 355 (94.7)	625 809 (95.6)	25 010 (99.3)	167 345 (94.1)
No	39 351 (4.6)	10 699 (5.3)	28 832 (4.4)	171 (0.7)	10 528 (5.9)

Abbreviations: CAHPS, Consumer Assessment of Healthcare Providers and Systems; MA, Medicare Advantage.

^a^ Unless otherwise specified, values are presented as the No. (%) of beneficiaries.

^b^ The MA integration dataset was merged to the Medicare Beneficiary Summary File and MA CAHPS data. Integrated MA plan beneficiaries were enrolled in an MA plan in January of the year of or after integration between MA plan and health system. Legacy- and non–legacy-integrated MA plan beneficiaries have a denominator of beneficiaries in integrated MA plans overall.

^c^ Includes any other race or ethnicity not included in the options listed.

Associations of care experiences with integrated and nonintegrated MA plans are reported in the Figure, A. Compared with being in a nonintegrated MA plan, being in an integrated MA plan was associated with small but significantly better mean ratings of customer service (1.6 points [95% CI, 1.1-2.1]) and health plan (1.0 points [95% CI, 0.6-1.5]). Beneficiaries in integrated MA plans reported significantly higher mean ratings of getting appointments and care quickly (0.8 points [95% CI, 0.3-1.3]), physicians who communicate well (0.6 points [95% CI, 0.3-0.8]), care coordination (0.5 points [95% CI, 0.2-0.8]), health care quality (0.5 points [95% CI, 0.2-0.8]), and drug plan (0.6 points [95% CI, 0.2-1.1]); however, these differences are likely too small to be meaningful for beneficiaries. Integrated MA plans were associated with substantially lower mean ratings of getting needed prescription drugs (−1.8 points [95% CI, −2.4 to −1.2]). No association was observed between integrated MA plans and mean ratings of getting needed care (0.1 points [95% CI, −0.4 to 0.5]). The results remained consistent in the sensitivity analysis (eTable 1 in Supplement 1).

**Figure.**
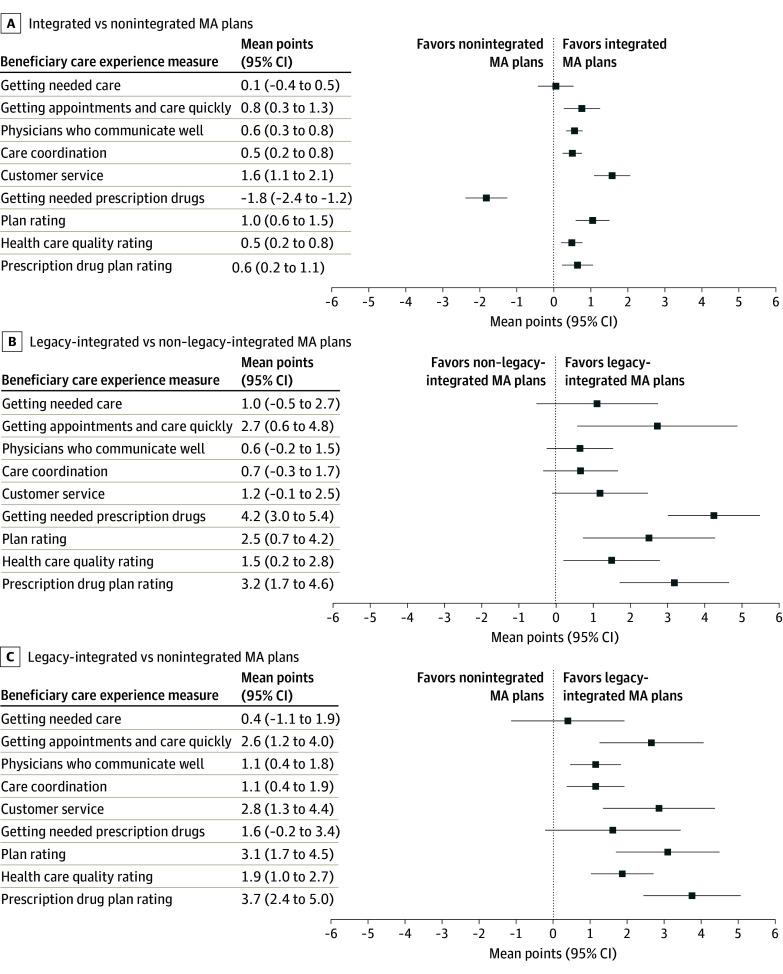
Care Experiences Among Medicare Advantage (MA) Beneficiaries by Plan Type A to C, Adjusted mean differences in beneficiary care experience measures between integrated MA plans and nonintegrated MA plans (reference) (A), legacy-integrated MA plans and non–legacy-integrated MA plans (reference) (B), and legacy-integrated MA plans and nonintegrated MA plans (reference) (C). Respondent-level mixed linear regressions were adjusted for age, race and ethnicity, sex, dual eligibility with Medicaid status, whether a proxy filled out the survey, health status, survey language, plan random effects, and state fixed effects. Values for means (95% CIs) for each outcome stratified by integration status were calculated using margins.

Legacy-integrated MA plans were associated with better care experiences than non–legacy-integrated MA plans across all measures (Figure, B). Compared with non–legacy-integrated MA plans, legacy-integrated MA plans were associated with better mean ratings of getting needed prescription drugs (4.2 points [95% CI, 3.0-5.4]), drug plan (3.2 points [95% CI, 1.7-4.6]), getting appointments and care quickly (2.7 points [95% CI, 0.6-4.8]), health plan (2.5 points [95% CI, 0.7-4.2]), and health care quality (1.5 points [95% CI, 0.2-2.8]). No association was observed between legacy-integrated MA plans and mean ratings of getting needed care (1.0 points [95% CI, −0.5 to 2.7]), physicians who communicate well (0.6 points [95% CI, −0.2 to 1.5]), care coordination (0.7 points [95% CI, −0.3 to 1.7]), and customer service (1.2 points [95% CI, −0.1 to 2.5]) compared with non–legacy MA plans.

Legacy-integrated MA plans were also associated with better care experience across all measures than nonintegrated MA plans (Figure, C). Compared with nonintegrated MA plans, legacy-integrated MA plans were associated with better mean ratings of drug plan (3.7 points [95% CI, 2.4-5.0]), health plan (3.1 points [95% CI, 1.7-4.5]), customer service (2.8 points [95% CI, 1.3-4.4]), getting appointments and care quickly (2.6 points [95% CI, 1.2-4.0]), health care quality (1.9 points [95% CI, 1.0-2.7]), physicians who communicate well (1.1 points [95% CI, 0 0.4-1.8]), and care coordination (1.1 points [95% CI, 0.4-1.9]). No association was observed between legacy-integrated MA plans and mean ratings of getting needed prescription drugs (1.6 points [95% CI, −0.2 to 3.4]) or getting needed care (0.4 points [95% CI, −1.1 to 1.9]) compared with nonintegrated MA plans.

## Discussion

In this cross-sectional analysis of CAHPS data, integrated MA plans overall were associated with small but meaningfully higher ratings of only customer service and health plan. All other associations with care experience measures were either too small to be meaningful or lower than nonintegrated MA plans. Legacy-integrated MA plans were associated with better ratings of all care experience measures compared with both non–legacy and nonintegrated MA plans. One of the primary arguments in favor of integrated MA plans is that integration will lead to better care experiences; however, our findings suggest that although integrated MA plans were associated with better care experiences, the associations were unlikely to be meaningful for beneficiaries unless they were in a legacy-integrated MA plan.

Previous studies reported that integrated MA plans are associated with higher quality ratings and more supplemental benefits, which likely represent the marginally better care experience in our findings.^3,7,20^ The association between vertical integration in MA and beneficiary care experience needs to be put into context with other literature. For beneficiaries, premiums were higher in integrated MA plans compared with nonintegrated MA plans.^3,7^ For the overall health care system, MA plans that were more integrated with practitioners had higher rates of upcoding compared with MA plans that were not as integrated with practitioners, which can lead to billions in overpayments.^27^ Given the financial consequences to beneficiaries and the health care system, more scrutiny is needed to assess whether integrated MA plans are meeting their potential.

More research is needed to understand what legacy-integrated MA plans may be doing to improve beneficiary care experience. There may be unmeasured differences of beneficiaries among legacy-integrated, non–legacy-integrated, and nonintegrated MA plans that are confounding the findings. In our sample, a higher proportion of beneficiaries in legacy-integrated MA plans were White, were nondual-eligible, did not need a proxy to complete the survey, reported good health status (excellent, very good, or good), and used English as the survey language compared with both non–legacy-integrated and nonintegrated MA plans, which suggests healthier beneficiaries. Considering the differences within integrated MA plans, future studies on vertical integration in MA should consider examining not only comparisons with beneficiaries in nonintegrated MA plans but also differences within integrated MA plans. There has been a call for more within MA research in general, because of concerns of confounding by unmeasured beneficiary differences.^28^

### Limitations

Our study has several limitations. First, unmeasured confounding may account for the association between vertical integration and beneficiary outcomes. Second, although we identified systems integrated vertically with health plans using a careful review of a range of different sources, there is some potential for misclassification of integrated systems. Third, there may be important heterogeneity in the integration between different contracts that we were unable to measure in this study, because there are no national data that collect and classify the forms of integration. Future work should develop validated measures of integration level to explore heterogeneity further. Legacy-integrated MA plans may carry a brand name (eg, Kaiser Permanente) to which beneficiaries assign their care experience, unlike beneficiaries in lesser-known integrated MA plans, which may bias the results. However, integrated MA plans consistently advertise their integration with a health system, so even beneficiaries in lesser-known integrated MA plans are likely to be aware of the integration and to assign their care experience to the entire system. Fourth, we also observed differences in CAHPS survey response rates, with 47.8% of respondents in legacy-integrated MA plans, 41.9% in non–legacy-integrated plans, and 38.6% in nonintegrated plans, which may bias our results (eTable 2 in Supplement 1). The confounding from differences in respondents is likely due to the same differences in types of beneficiaries enrolled; therefore, although we attempted to adjust for measured confounders, it is likely that some unmeasured confounding may still be biasing the results and further suggest purposeful enrollment differences. Despite these limitations, this is the first study, to our knowledge, to evaluate the association of health system with insurance vertical integration and care experiences in the US.

## Conclusions

The findings of this cross-sectional study suggest that integrated MA plans are not associated with a meaningfully better beneficiary care experience for nearly all measures examined compared with nonintegrated MA plans. The association between enrollment in legacy-integrated MA plans and meaningfully better care experiences on all measures compared with non–legacy integrated and nonintegrated MA plans suggests that vertical integration may take longer to affect beneficiaries or these plans may enroll a different type of beneficiary. Monitoring of integrated MA plans is needed to assess whether they are adding meaningful value to MA beneficiaries and to determine their effects on the health care system.

## Notes

Supplement 1.
eMethods
**eTable 1.** Sensitivity Analysis Removing Kaiser Permanente Beneficiaries **eTable 2.** Survey Respondents by Integration Status

Supplement 2.
Data Sharing Statement
