# Supplementary material for: Vertical Integration and Care Experiences Among Medicare Advantage Beneficiaries
Source: JAMA Netw Open. 2024 Oct 17;7(10):e2438994. doi: 10.1001/jamanetworkopen.2024.38994 (PMC11581604; doi:10.1001/jamanetworkopen.2024.38994)
Supplement: Supplement 1. — eMethods eTable 1. Sensitivity Analysis Removing Kaiser Permanente Beneficiaries eTable 2. Survey Respondents by Integration Status [file jamanetwopen-e2438994-s001.pdf]

Supplementary Online Content

Bejarano G, Ryan A, Trivedi A, Meyers DJ. Vertical integration and care experiences among Medicare Advantage beneficiaries. *JAMA Netw Open*. 2024;7(10):e2438994. doi:10.1001/jamanetworkopen.2024.38994

eMethods

eTable 1. Sensitivity Analysis Removing Kaiser Permanente Beneficiaries

eTable 2. Survey Respondents by Integration Status

This supplementary material has been provided by the authors to give readers additional information about their work.

© 2024 Bejarano G et al. *JAMA Network Open.*

eMethods

Summary of vertical Integration dataset and MA CAHPS composite measures

Vertical Integration Definition
For each MA contract, we assessed vertical integration status through a combination of the contract's website and marketing materials, publicly available tax documents, and news reports. For each contract we also reported what year the integration occurred either as the year the contract was formed if integrated from inception or the first reported merger between the contract and a provider. After identifying each integrated contract, we used marketing materials and tax documents to further compile a list of all provider IDs that are included in the system (primarily CMS CCN IDs and TINs). We assigned each combination of providers and plans with a unique system ID for use in our dataset. When assessing the integration of contracts, we classified them into three categories: 1) full health system integrations (where the same business entity owns both the plan and a health system that includes a hospital), 2) full provider organization integrations (where a single financial entity owns an outpatient provider group but does not include a hospital), 3) financially aligned systems (where the same company did not own both the plan and provider, but when there was a strong financial arrangement between the two). To validate the dataset, we compared our list of integrated plans and providers against several different sources including a Becker Hospital Review list, the AHRQ Compendium of US Health Systems, and other published work that traces the growth of integrated MA contracts over time.

MA CAHPS Outcome Measures
All 6 composite and 3 single-item measures were converted to a 0-100 scale. The composite measures are listed below:
Getting Needed Care:
- In the last 6 months, how often was it easy to get the care, tests or treatment you needed? (never, sometimes, usually, always)
- In the last 6 months, how often did you get an appointment to see a specialist as soon as you needed? (never, sometimes, usually, always)

Getting Appointments and Care Quickly:
- In the last 6 months, when you needed care right away, how often did you get care as soon as you needed? (never, sometimes, usually, always)
- In the last 6 months, how often did you get an appointment for a check-up or routine care as soon as you needed? (never, sometimes, usually, always)

Physicians Who Communicate Well
- In the last 6 months, how often did your personal doctor explain things in a way that was easy to understand? (never, sometimes, usually, always)
- In the last 6 months, how often did your personal doctor listen carefully to you? (never, sometimes, usually, always)
- In the last 6 months, how often did your personal doctor show respect for what you had to say? (never, sometimes, usually, always)
- In the last 6 months, how often did your personal doctor spend enough time with you? (never, sometimes, usually, always)

Care Coordination
- In the last 6 months, when you visited your personal doctor for a scheduled appointment, how often did he or she have your medical records or other information about your care? (never, sometimes, usually, always)
- In the last 6 months, when your personal doctor ordered a blood test, x-ray or other test for you, how often did someone from your personal doctor's office follow up to give you those results? (never, sometimes, usually, always)
- In the last 6 months, when your personal doctor ordered a blood test, x-ray or other test for you, how often did you get those results as soon as you needed them? (never, sometimes, usually, always)
- In the last 6 months, how often did you and your personal doctor talk about all the prescription medicines you were taking? (never, sometimes, usually, always)
- In the last 6 months, did you get the help you needed from your personal doctor's office to manage your care among these different providers and services? (never, sometimes, usually, always)

© 2024 Bejarano G et al. *JAMA Network Open.*

- In the last 6 months, how often did your personal doctor seem informed and up-to- date about the care you got from specialists? (never, sometimes, usually, always)

Customer Service

- In the last 6 months, how often did your health plan's customer service give you the information or help you needed? (never, sometimes, usually, always)
- In the last 6 months, how often did your health plan's customer service staff treat you with courtesy and respect? (never, sometimes, usually, always)
- In the last 6 months, how often were the forms from your health plan easy to fill out? (never, sometimes, usually, always)

Getting Needed Prescription Drugs

- In the last 6 months, how often was it easy to use your prescription drug plan to get the medicines your doctor prescribed? (never, sometimes, usually, always)
- In the last 6 months, how often was it easy to use your prescription drug plan to fill a prescription at your local pharmacy? (never, sometimes, usually, always)
- In the last 6 months, how often was it easy to use your prescription drug plan to fill a prescription by mail? (never, sometimes, usually, always)

eTable 1. Sensitivity Analysis Removing Kaiser Permanente Beneficiaries

Adjusted average beneficiary care experience measures between integrated MA plans compared to non-integrated MA plans

Outcome	Integrated MA plan, mean(95% CI)	Non-integrated MA plan, mean(95% CI)	Mean difference (95% CI)	p-value
Getting needed care	84.6 (83.9 to 85.2)	84.5 (84.0 to 85.1)	0.02 (-0.5 to 0.5)	0.926
Getting appointments and care quickly	77.8 (77.0 to 78.6)	77.2 (76.5 to 77.9)	0.6 (0.1 to 1.1)	0.014
Physicians who communicate well	91.3 (91.0 to 91.6)	90.8 (90.6 to 91.1)	0.5 (0.3 to 0.7)	<0.001
Care coordination	69.8 (69.4 to 70.2)	69.3 (69.0 to 69.6)	0.5 (0.2 to 0.7)	0.001
Customer service	85.0 (84.5 to 85.5)	83.5 (83.2 to 83.9)	1.5 (1.0 to 2.0)	<0.001
Getting needed prescription drugs	62.6 (62.0 to 63.2)	64.7 (64.4 to 65.1)	-2.1 (-2.7 to -1.5)	<0.001
Rating of health plan	86.0 (85.5 to 86.5)	85.1 (84.8 to 85.5)	0.9 (0.4 to 1.3)	<0.001
Rating of health care quality	85.9 (85.6 to 86.3)	85.6 (85.3 to 85.9)	0.4 (0.08 to 0.7)	0.011
Rating of drug plan	85.4 (84.9 to 85.9)	85.1 (84.7 to 85.4)	0.4 (-0.05 to 0.8)	0.085

© 2024 Bejarano G et al. *JAMA Network Open.*

eTable 2. Survey Respondents by Integration Status

Characteristic		Legacy Integrated MA plan No. (%)	Non-Legacy Integrated MA plan No. (%)	Non-Integrated MA plan No. (%)
Survey Responder	Yes	25,695 (47.8)	183,600 (41.9)	672,762 (38.6)
	No	28,081 (52.2)	254,508 (58.1)	1,070,110 (61.4)

© 2024 Bejarano G et al. *JAMA Network Open.*
