# Supplementary material for: Vertical Integration and Care Experiences Among Medicare Advantage Beneficiaries
Source: JAMA Netw Open. 2024 Oct 17;7(10):e2438994. doi: 10.1001/jamanetworkopen.2024.38994 (PMC11581604; doi:10.1001/jamanetworkopen.2024.38994)
Supplement: Supplement 2. — Data Sharing Statement [file jamanetwopen-e2438994-s002.pdf]

Data Sharing Statement

Bejarano. Vertical Integration and Care Experiences Among Medicare Advantage Beneficiaries. *JAMA Netw Open*. Published October 17, 2024. doi:10.1001/jamanetworkopen.2024.38994

Data
Data available: No

Additional Information
Explanation for why data not available: Data used in this study were granted under a data-use agreement with the US Centers for Medicare & Medicaid Services and cannot be provided without its consent. Code used in the calculation of these results are available from the corresponding author on request.
